# Viral shedding in patients with hand, foot and mouth disease induced by EV71, CA16, or CA6: A protocol for systematic review and meta analysis

**DOI:** 10.1097/MD.0000000000021258

**Published:** 2020-07-17

**Authors:** Xianzhi Li, Qiuxia Wang, Zhenhua Chen, Xiaoxia Duan, Yutong Han, Rongsheng Luan, Lu Long

**Affiliations:** a Department of Epidemiology and Health Statistics, West China School of Public Health and West China Fourth Hospital, Sichuan University, Chengdu; b Department of Radiology, Tongji Hospital, Tongji Medical College, Huazhong University of Science and Technology, Wuhan; c Department of Microbiology Laboratory, Chengdu Municipal Center for Disease Control and Prevention, Sichuan, China.

**Keywords:** hand, foot, and mouth disease, enterovirus, isolation period, single-arm meta-analysis, systematic review, viral shedding

## Abstract

**Introduction:**

Hand, foot, and mouth disease (HFMD) has been an important public health concern worldwide, especially in the Asia-Pacific region. Unfortunately, the effect of current measures on preventing and controlling HFMD may be limited. Isolation of infectious sources is reported as an important way to prevent and control this disease. The isolation period is determined on the basis of duration of viral shedding in patients with HFMD. However, the results of previous researches on duration of viral shedding remain controversial. Here, we present a protocol of a systematic review and single-arm meta-analysis for assessing the duration of viral shedding in patients with HFMD induced by Enterovirus 71 (EV71), Coxsackievirus A16 (CA16), or Coxsackievirus A6 (CA6).

**Methods and analysis:**

A comprehensive literature search will be performed in PubMed, EMBASE, the Cochrane library, Chinese National Knowledge Infrastructure (CNKI), Chinese Biomedical Literature Database (CBM), and Wanfang Database, covering the period from inception to May 1, 2019. Point estimate of positive rate with corresponding 95% confidence intervals (CIs) of EV71, CA16, or CA6 in HFMD patients’ fecal or throat samples will be carried out using STATA 14.0. Subgroup analyses will be performed for mild cases, severe cases, and close contacts. Sensitive analysis will also be performed to evaluate the influences of individual studies on the final effect by exclusion of a few articles of poor quality. We will assess the risk of bias for the final studies included in our meta-analysis using previously available tools and the modified risk of bias tool.

**Results:**

The results of this systematic review and meta-analysis will be published in a peer-reviewed journal.

**Conclusion:**

To the best of our knowledge, this paper will be the first systematic review and meta-analysis for assessing the duration of viral shedding in patients with HFMD induced by EV71, CA16, or CA6. The conclusions drawn from this review will provide the scientific basis to formulate the isolation period of HFMD.

**Ethics and dissemination:**

Ethical review is not required as this article is for a systematic review since there is no direct involvement of patients in the whole process. We will publish the results of this systematic review and meta-analysis of single-arm studies in a peer-reviewed journal.

**Registration number:**

Prospero CRD42020139999.

**Key points**

(a) To the best of our knowledge, this is the first systematic review and meta-analysis for assessing the duration of viral shedding in patients with HFMD induced by EV71, CA16, or CA6.
(b) Designing different isolation period according to major routes of transmission in a certain region, and our results will make this strategy possible.
(c) Our results will also provide support to design diverse isolation period for patients infected with different enterovirus serotypes and patients with different severity of HFMD.
(d) Different time of patients’ samples are collected after onset, storage condition of samples, proficiency of sample testers may run the risk of heterogeneity.
(e) The proportion of loss to follow-up is not relatively low in some included study.

## 1 Introduction

Hand, foot, and mouth disease (HFMD) is a contagious viral illness that commonly affects infants and children under 5 years of age.^[1]^ Enterovirus 71 (EV71), Coxsackievirus A16 (CA16), and Coxsackievirus A6 (CA6) are generally considered as the most common causative pathogens for HFMD, while EV71 is the most frequently identified serotype among severe and fatal cases.^[2–4]^ Over the last decade, with the increasing frequency and severity of the outbreak, HFMD has been an important public health concern worldwide, especially in the Asia-Pacific region.^[5–7]^ Currently, there is no specific antiviral drug available for this disease.^[2]^ The inactivated monovalent EV71 vaccine was licensed in China in 2015,^[8]^ which is highly effective against EV71-associated HFMD but no cross-protection against HFMD caused by other serotypes in children.^[9–11]^ Although the HFMD cases induced by EV71 were significantly decreased over the past several years, the prevalence of HFMD induced by other serotypes remain high in China,^[12]^ which suggests the effect of current measures on preventing and controlling HFMD may be limited.^[13,14]^

According to Guide of Prevention and Control for HFMD in China, the isolation period, which is determined based on duration of viral shedding in patients with HFMD, is from the onset to 1 week after the clinical symptoms disappear.^[15,16]^ However, the results of previous researches on duration of viral shedding remain controversial. A study from mainland China presented that EV71 could be detected by using a nested RT-PCR (Reverse Transcription-Polymerase Chain Reaction) up to almost 3 weeks after onset for throat swabs, and 7 weeks after onset for fecal specimens.^[13]^ Another study in Taiwan, where viral isolation was used to detect EV71, found that EV71 were still detectable in respiratory samples sixth weeks after onset and in feces more than 11 weeks.^[17]^ Besides, the duration of enterovirus shedding was reported to last for a longer period in fecal samples than that in throat swabs, and be associated with enterovirus serotypes.^[13,17,18]^

The reasons for the inconsistency of results about viral shedding from various studies may be due to small sample size, loss of follow-up, different enterovirus serotypes, the type of detection samples, the severity of disease, and virus detection methods.^[13,14,16,18–21]^ Recognizing that individual studies might not be able to provide sufficient data on their own to formulate the isolation period of HFMD, we will conduct a single-arm meta-analysis of evidence from studies of the duration of viral shedding in patients with hand, foot and mouth disease induced by EV71, CA16, or CA6 to provide the scientific basis for control and prevention of HFMD.

## 2 Objectives

The objectives of this study are as follows:

1. We will conduct a single-arm meta-analysis of evidence from studies of the duration of viral shedding in patients with HFMD induced by EV71, CA16, or CA6 to provide the scientific basis for control and prevention of HFMD;
2. Furthermore, in subgroup analyses, we will compare the duration of EV71, CA16, or CA6 shedding in stool and throat specimens from patients with different severity of HFMD, including close contacts, mild cases and severe cases.

## 3 Methods

### 3.1 Standards and registration

The protocol was developed in accordance with the Preferred Reporting Items for Systematic Reviews and Meta-Analysis protocols (PRISMA-P) statements and has been registered with PROSPERO (CRD42020139999), an international prospective register of systematic reviews.

### 3.2 Search strategy

A comprehensive database search will be performed in PubMed, EMBASE, the Cochrane library, Chinese National Knowledge Infrastructure (CNKI), Chinese Biomedical Literature Database (CBM), and Wanfang Database, covering the period from inception to May 1, 2019. The following medical subject headings and keywords, hand-foot-mouth disease, herpangina, EV71, CA16, and CA6, and viral shedding, will be used to identify prospective study determining the duration of viral shedding in the throat or fecal samples of HFMD patients induced by the above three viruses. For details of the preliminary search strategy in PubMed see Table 1. All prospective studies published in English or Chinese will be included, and the reference lists in relevant review articles will be scanned manually. All identified articles will be screened independently by 2 reviewers.

**Table 1:**
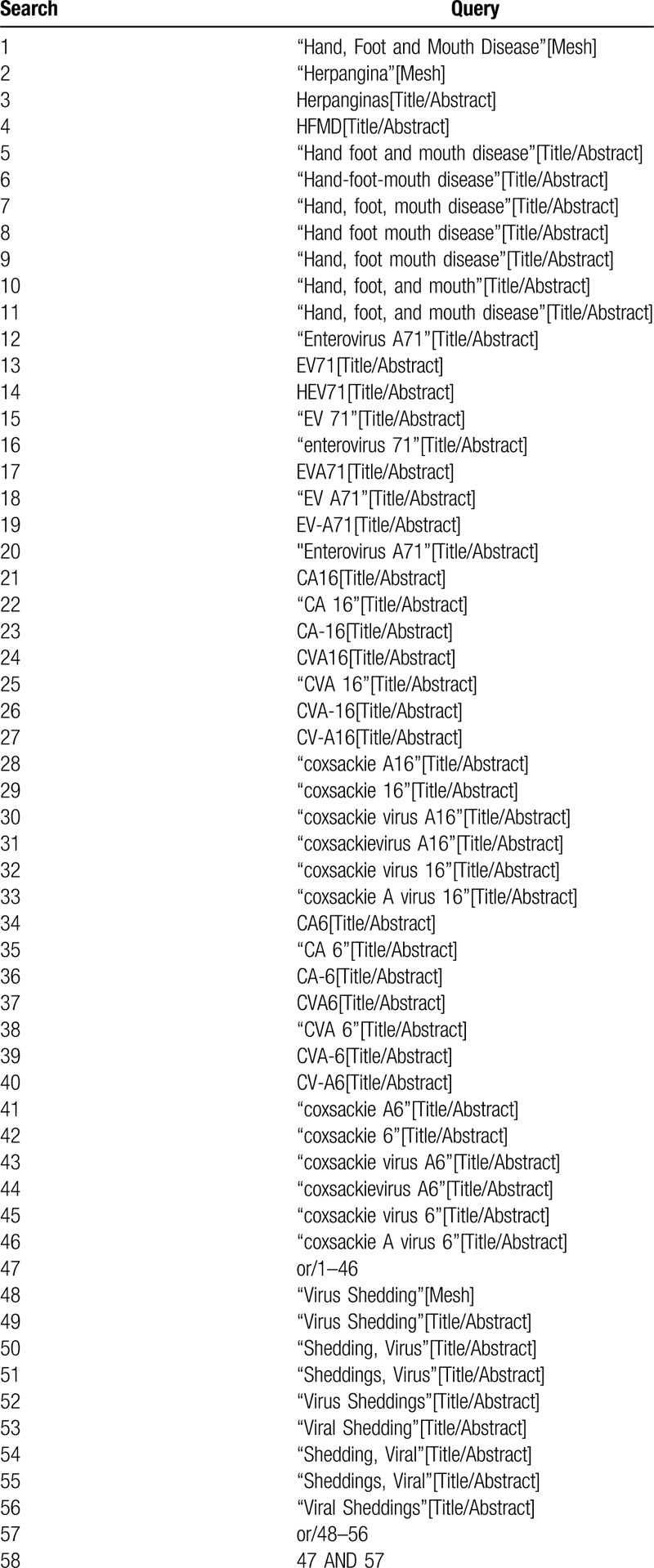
Preliminary search strategy: PubMed format.

### 3.3 Eligibility criteria

Publications meet the following criteria will be eligible:

(a) Research type: prospective study;
(b) Research subjects: children with a laboratory diagnosis of HFMD induced by EV71, CA16, or CA6 or their close contacts;
(c) Research purposes: to determine the duration of viral shedding in the throat or stool samples of HFMD patients;
(d) Follow-up time: Follow-up time of at least 1 weeks;
(e) Virus detection method: PCR or viral isolation;
(f) Accessible information: extractable basic data and literature information, and clear follow-up data including the number of people detected and the number of people who detected positive at each detection time point.

Publications meet one of the following criteria will not be eligible:

(a) Non-prospective study;
(b) The relevant information could not be extracted or follow-up data are not clear;
(c) Duplicate publication;
(d) The virus serotype that causes HFMD is unclear, or the virus serotype that causes HFMD is non-EV71, non-CA16, or non-CA6;
(e) HFMD patients are infected with two or more of the above three serotypes, but the duration of virus shedding of each serotype is unclear.

### 3.4 Case definition

According to Chinese Guidelines for the Diagnosis and Treatment of Hand, foot and mouth disease (2018 edition), the mild HFMD is defined as fever and rash on the hands, feet, mouth, or buttocks. And the severe HFMD is defined as mild HFMD with neurological, respiratory, or circulatory complications.^[22]^ Close contact is defined as people who spent a lot of time living with those confirmed HFMD cases.

### 3.5 Outcome measurement

The outcome of interest is the positive rates of the above three enterovirus serotypes in the throat or stool samples of HFMD patients. The rates are calculated as the number of patients detected positive for the virus divided by the total number of patients detected at a time point.

### 3.6 Data management

We will upload all the search results in one single EndNote (X9) library and remove duplicates.

### 3.7 Selection process

**Figure 1:**
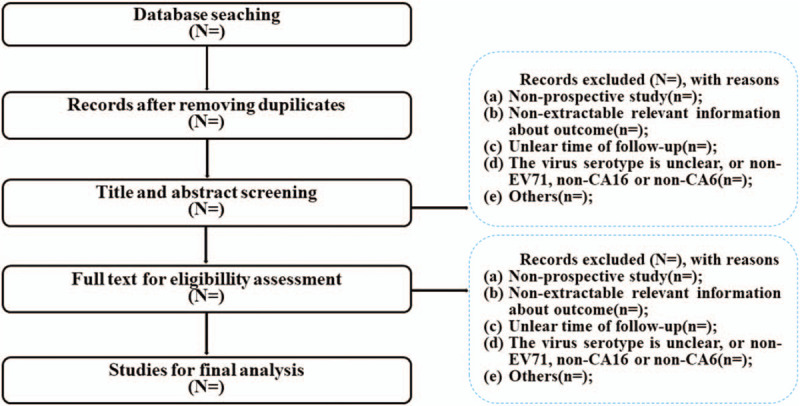
Flow chart of literature screening process.

We will review study titles and abstracts to determine studies that meet the inclusion criteria. Then, these studies meet the inclusion criteria will be further identified by reviewing the full-text articles. And reasons for the exclusion of articles in the process of screening will be documented. All works will be completed by two investigators independently and any discrepancies will be resolved by discussion. If consensus cannot be reached, disagreements will be resolved by a third investigator. A proposed flow chart is shown in Figure 1.

### 3.8 Data extraction

The following information will be extracted independently by 2 reviewers from the eligible papers meeting the inclusion criteria: author, year of publication, study design, number, and characteristics of the participants in study, enterovirus serotypes that causes HFMD (EV71, CA16, or CA6), the type of detection sample (throat or fecal swab), virus detection method (RT-PCR or viral isolation), the time of patients’ samples are collected after onset, the absolute number of patients detected positive for the virus serotype and the total number of patients detected. If the absolute number of patients detected positive for the virus serotype is not reported, we will estimate the number of patients detected positive for the virus serotype from the reported cumulative incidence and the total number of patients detected. If the absolute number and the cumulative incidence of the outcomes of interest are not reported, we will use the reported Kaplan–Meier curve to obtain the cumulative incidence. If the information we need is not still gained, we will ask the author for help by email.

### 3.9 Risk of bias assessment

Risk of bias for the final studies included in our meta-analysis will be assessed using previously available tools and the modified risk of bias tool,^[23–25]^ which assess bias in observational studies based on the following items:

(a) Sampling method: whether a probability sampling (e.g., Simple random sampling, stratified random sampling, cluster sampling, or systematic sampling) or non-probability sampling was used to select the sample;
(b) Response rate: whether the response rate was 80% or more;
(c) Definition: whether the HFMD induced by EV71, CA16, or CA6 was diagnosed by the laboratory test;
(d) Follow-up: whether patients were followed up until more than 80% of the patients tested negative for the enteroviruses;
(e) Data collection: whether data were collected from subjects directly or via proxy;
(f) Measurement: whether the identification of viral shedding was based on a laboratory test for pathogens;
(g) Precise parameter: whether the parameter of interest was the adjusted/stratified positive rate by the severity of disease.

### 3.10 Data synthesis

Meta-analysis will be performed separately according to the enterovirus serotypes, the type of detection sample, virus detection method, and the time of patients’ samples are collected after onset (1 week, 2 weeks, 3 weeks, and so on).

### 3.11 Statistical analyses

The pooled positive rates and 95% CIs (standard of test α = 0.05) will be calculated.

1. Heterogeneity evaluation of the literatures and analysis of pooled effect sizes: The Cochran *Q* test and *I*^2^ statistic will be used to evaluate heterogeneity. The *I*^2^ will be used to assess the heterogeneity as the following criteria: low at <25%, moderate at 25% to 50%, and high at >50%. Theoretically, if no significant heterogeneity exists, pooled estimates of positive rates will be calculated by a fixed-effects model; otherwise, a random-effects model will be used.
2. Subgroup analyses: We will perform subgroup analyses to investigate the duration of viral shedding in patients with HFMD induced by EV71, CA16, or CA6 by severity of disease, categorized as
  (a) mild cases
  (b) severe cases
  (c) close contacts
3. Sensitivity analysis: Sensitive analysis will also be performed to evaluate the influences of individual studies on the final effect by exclusion of a few articles of poor quality; this meta-analysis will be considered trusted if the result of meta-analysis is stable.
4. Analysis of publication bias: a funnel plot will be constructed to assess the possibility of publication bias. Then, Begg and Egger tests will be used to assess funnel plot asymmetry.

We define significant publication bias as a *P* value <.1. If publication bias exists, the trim-and-fill computation will be used to estimate the effect of publication bias on the interpretation of the results. All of the statistical analyses will be performed by STATA 14.0 (version 14.0, Stata corp., College Station, TX).

### 3.12 Ethics and dissemination

Ethical review is not required as this article is for a systematic review since there is no direct involvement of patients in the whole process. This study was funded by National Natural Science Foundation of China (grant number 81903375), Postdoctoral Research Foundation of China (grant number 2018M643509) and Fundamental Research Funds for the Central Universities (grant number 2017SCU12029). We will report our results comprehensively for peer-reviewed journal.

## 4 Discussion

This protocol presents the methodology of a systematic review and meta-analysis for assessing the duration of viral shedding in patients with HFMD induced by EV71, CA16, or CA6. The conclusions drawn from this review will provide the scientific basis to formulate the isolation period of HFMD.

Routes of transmission of HFMD are multiple. In areas with poor sanitary conditions, fecal-oral transmission may predominate, whereas in areas with good sanitary conditions, respiratory transmission may be more important.^[26]^ So, it is reasonable to design different isolation period according to major routes of transmission in a certain region, and our results will make this strategy possible. Besides, our results will also provide support to design diverse isolation period for patients infected with different enterovirus serotypes and patients with different severity of HFMD. Overall, we believe that our systematic assessment will provide important guiding significance for the prevention and control of HFMD.

There may be some limitations in this review. First, our assessment will only include studies published in English and Chinese. Because of the barrier of language, relevant studies in some other languages may be missed. Also, different time of patients’ samples are collected after onset, storage condition of samples, proficiency of sample testers may run the risk of heterogeneity. Finally, the proportion of loss to follow-up is not relatively low in some included study, which may influence our quality of this assessment.

## Author contributions

XZL, QXW, and LL contributed to the conception, design and preliminary search of the systematic review and meta-analysis. XZL drafted this protocol. XZL, LL, ZHC, RSL, YTH, and XXD provided critical revisions of the protocol and approved submission of the final manuscript.
